# Exosomes as Biomarkers for Female Reproductive Diseases Diagnosis and Therapy

**DOI:** 10.3390/ijms22042165

**Published:** 2021-02-22

**Authors:** Sahar Esfandyari, Hoda Elkafas, Rishi Man Chugh, Hang-soo Park, Antonia Navarro, Ayman Al-Hendy

**Affiliations:** 1 Department of Surgery, University of Illinois at Chicago, Chicago, IL 60612, USA; sesfan2@uic.edu (S.E.); helkaf2@uic.edu (H.E.); rchugh@kumc.edu (R.M.C.); 2 Department of Physiology and Biophysics, University of Illinois at Chicago, Chicago, IL 60612, USA; 3 Department of Pharmacology and Toxicology, Egyptian Drug Authority (EDA) Formally, (NODCAR), Cairo 35521, Egypt; 4 Department of Radiation Oncology, University of Kansas Medical Center, Kansas City, KS 66160, USA; 5 Department of Obstetrics and Gynecology, University of Chicago, Chicago, IL 60637, USA; hspark06@bsd.uchicago.edu (H.-s.P.); anavarr1@bsd.uchicago.edu (A.N.)

**Keywords:** Exosome, infertility, female reproductive diseases

## Abstract

Cell–cell communication is an essential mechanism for the maintenance and development of various organs, including the female reproductive system. Today, it is well-known that the function of the female reproductive system and successful pregnancy are related to appropriate follicular growth, oogenesis, implantation, embryo development, and proper fertilization, dependent on the main regulators of cellular crosstalk, exosomes. During exosome synthesis, selective packaging of different factors into these vesicles happens within the originating cells. Therefore, exosomes contain both genetic and proteomic data that could be applied as biomarkers or therapeutic targets in pregnancy-associated disorders or placental functions. In this context, the present review aims to compile information about the potential exosomes with key molecular cargos that are dysregulated in female reproductive diseases which lead to infertility, including polycystic ovary syndrome (PCOS), premature ovarian failure (POF), Asherman syndrome, endometriosis, endometrial cancer, cervical cancer, ovarian cancer, and preeclampsia, as well as signaling pathways related to the regulation of the reproductive system and pregnancy outcome during these pathological conditions. This review might help us realize the etiology of reproductive dysfunction and improve the early diagnosis and treatment of the related complications.

## 1. Introduction

Intercellular interaction is a critical factor in organizing cellular events in all organisms. In multicellular organisms, various strategies have been implied in cellular cross-talk and cell–cell communication. These strategies are either direct interplay by gap junctions or an indirect mechanism in which secreted extracellular signals are involved [1]. The second procedure follows different stages, such as the production and secretion of hormones, cytokines, and growth factors into the extracellular spaces and their attachment to the target cells for influencing target cells’ activities. The transmission of hormones in the circulation system is the most prominent example of intercellular interaction resulting in specific signaling pathways among cells [2]. Mainly, cells in the reproductive tissues are in constant communication affected by autocrine (the same cell generating the signal), endocrine (between distant cells), and paracrine (between nearby cells) signaling pathways [3,4]. 

Several studies have recently suggested extracellular vesicles (EVs) as a new mechanism mediating cellular crosstalk within or among tissues [5,6,7]. Moreover, the use of EVs in diagnosis and treatment has been indicated in many studies. EVs are an interesting subject in reproductive disorder therapeutics because of their ability to transfer various molecules either in normal or abnormal conditions [8]. EVs are a heterogeneous population of nanoparticles secreted by different cells in an evolutionarily conserved manner [9,10]. This shuttling activity happens through at least two mechanisms: (i) by the receptor/ligand interaction between EVs and target cells or (ii) by attachment of EVs with target plasma cells following the membrane/membrane fusion with the transfer of their content within the target cells [8,11,12]. Therefore, these vesicles contain bioactive molecular signals, including DNAs, RNAs (both coding and non-coding RNAs), lipids, and proteins, as well as chemical compounds like drugs, including Cisplatin, Doxorubicin, Curcumin, and Acridine Orange, which mediate target cells’ functions by either induction of surface ligands or transferring factors associated with different biological pathways [9,13,14]. Nevertheless, the EVs role in cell–cell communication is highly influenced by the microenvironment they are present in, for example, the pH. In this case, it was reported that there was a higher release and uptake of EVs at lower pH in contrast to a buffered condition [11,15]. Furthermore, along with the rapid growth of epigenetic investigations, there is great evidence that circulating EVs might transfer information as foreign genes to germinal cells. In other words, it was indicated that somatic nucleic acids were transferred to germinal cells by EVs, which subsequently acted as the final recipients of somatic cell-derived data [16].

There are different types of EVs, including exosomes, microvesicles (MVs), apoptotic bodies (ABs) according to their size, specific surface markers, biogenesis, and content [17,18]. Among them, exosomes are known as effective paracrine regulators of cellular crosstalk and are present in various biological fluids. Different functions have been identified for these molecules, including metabolism regulation, cell proliferation, apoptosis, angiogenesis, antigen presenting, inflammatory pathways, tumor pathogenesis, tissue repair, and reproduction [19,20,21].

**Figure 1:**
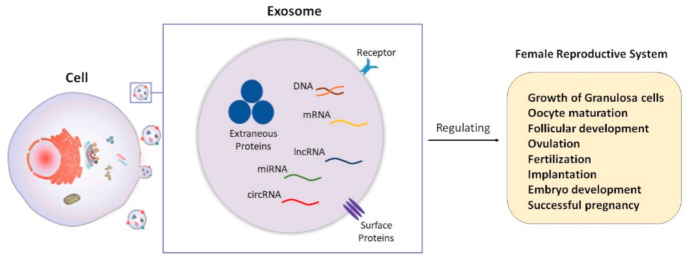
A schematic of the role of exosomes in different biological activities of female reproductive system.

Exosome secretion has been addressed in a variety of reproductive cells, such as endometrial cells [22], follicular cells [5], embryos produced in vitro [23], and oviductal cells [6]. Indeed, the function of the female reproductive system and successful pregnancy have a remarkable association with appropriate follicular growth, oogenesis, implantation, embryo development, and proper fertilization, which are affected by intercellular communication, as well as the interaction between mother and embryo during pregnancy (Figure 1) [21,22,24]. Furthermore, numerous reproductive pathological mechanisms are linked to the exosomes’ spread in body fluids [5,6,7,25,26]. Recent literature indicated that exosomes are synthesized and released from different parts of the female reproductive tract, including oviductal epithelium, follicular fluid, endometrium, uterine, embryos in culture media, and the placenta [27,28,29,30]. 

Several studies on the exosomal component profiles in human samples and different animal models implied that exosomes transfer specific molecular cargos, in particular, microRNAs (miRNAs), which could target signaling pathways associated with meiotic resumption, follicular development, oocyte maturation, embryo development, and ovulation. Indeed, it was suggested that in these districts, a remarkable number of miRNAs are transferred by exosomes and thus, cannot simply flow through follicular fluid and plasma like various follicle compartments [31,32]. miRNAs are a large group of 21–24 nucleotides non-coding RNAs linked to numerous biological activities [33] and are necessary for oocyte-specific pathways, including oocyte maturation, implantation, and early embryonic development during the oocyte developmental competence and follicular growth by targeting genes involved in related pathways [34]. Among these, the most affected signaling pathways regulated by these factors are insulin, wingless (Wnt), mitogen-activated protein kinase (MAPK), neurotrophin, epidermal growth factor receptor (ErbB), and transforming growth factor-beta (TGF-β). Moreover, even molecules linked to ubiquitin-mediated pathways inside exosomes can be controlled by some miRNAs [35,36,37].

**Figure 2:**
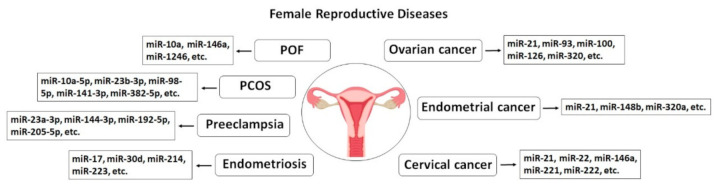
A summary of exosomal miRNAs reported in recent studies considering their role in the pathogenesis of the most noticeable female reproductive diseases. miR, miRNA, microRNA; POF; Premature Ovarian Failure, PCOS; Polycystic Ovary Syndrome.

In this context, exosomes transport different cargos and, thus, play an important role in the regulation of gene and protein expression, proliferation and differentiation of granulosa cells and follicles, oocyte growth, fertilization, implantation, embryo development, and successful pregnancy [38,39]. Due to exosomes’ diagnostic and therapeutic potential in reproductive disorders, great attention has shifted toward the role of exosomes over the last two decades [40]. Hence, in this review, we will discuss the involvement of exosomes and their important cargos, such as miRNAs, in the progression of different reproductive disorders, including polycystic ovary syndrome (PCOS), premature ovarian failure (POF), Asherman syndrome, endometriosis, endometrial cancer, cervical cancer, ovarian cancer, and preeclampsia (Figure 2). However, we will first briefly describe their characterization, formation, and function in the female reproductive system. This review might help us realize the etiology of reproductive dysfunction and improve the early diagnosis and treatment of related complications.

## 2. Exosomes Biogenesis, Components, and Characterization

Exosomes are a type of lipid bilayer membrane vesicle secreted by cells in the extracellular spaces and respond to particular stimulus in physiological or pathological circumstances [41,42,43]. These particles are a subtype of EVs typically 30–150 nm in diameter [44]. They contain constituents, including DNAs and RNAs [45], proteins [46], and lipids [47], and can exchange them between cells. These components imply the cell regulating activities of exosomes. Remarkably, exosome-mediated nucleic acid transfer between cells leads to a new “genetic exchange” process [48].

The biogenesis of exosomes begins with an endosomal process in a stepwise pathway. It initiates via endocytosis, or the inside budding development of cell membranes, resulting in the formation of endocytic vesicles with inverted lipid bilayer membranes that finally produce early endosomes. In the next step, the early endosomes enter two different pathways, ultimately creating either late endosomes or recycling endosomes [49]. The early endosomes, undergoing a series of pathways such as inward budding of endosomal membranes, form the late endosomes or, in other words, the multivesicular bodies (MVBs). MVBs could both combine with lysosomes to lyse their components or combine with the plasma membrane to produce intraluminal vesicles (ILVs) with a diameter of 30–100 nm. Then, these ILVs in the extracellular environment are generally recognized as exosomes [50,51]. This process was introduced for the first time during in vitro maturation of reticulocytes [52,53]. On the other hand, the early endosomes, which combine with other endocytic vesicles, create the recycling endosomes. These types of endosomes transfer molecules into the plasma membrane for recycling [49].

Exosomes communicate with the target cells via ligand-receptor interaction, fusion, or internalization through receptor-induced endocytosis [54]. The underlying mechanism of exosomes and target cell interplay is followed, as the interaction between exosome membrane proteins induces intracellular signaling pathways within the recipient cells. In the next steps, the cleavage of the exosomal membrane proteins near the receptors of recipient cells occurs, the exosome components are transferred into cells, and these vesicles enter via phagocytosis [42,55]. There are numerous common functional proteins, such as β-actin; GPI-anchored proteins; heat shock protein 8 (Hsp8); Hsp90; tubulin; and the tetraspanins CD9, CD63, and CD81. Exosomes could also be characterized by these membranous and cytoplasmic proteins [56].

It should be noted that exosomes are secreted in both physiological and pathological situations. It is suggested that exosome contents reflect the status of their donor cells. In this context, according to the cell pathophysiological status, exosomes may represent particular components. This feature of exosomes makes them applicable prognostic and diagnostic molecules for various disorders. However, we should consider that the collection and investigation of exosomes is still a challenge due to the lack of precise methods for isolation [57,58]. Remarkably, recent strides collected several exosomes from extracellular fluids derived from both culture media and body fluids. Therefore, they seem to be practical molecules for clinical studies, diagnostic procedures, and therapeutic approaches [58]. 

## 3. Exosomes in the Pathophysiology of Female Reproduction

In this context, exosomes transport different cargos and, thus, play an important role in the regulation of gene and protein expression, proliferation and differentiation of granulosa cells and follicles, oocyte growth, fertilization, implantation, embryo development, and successful pregnancy [59,60]. Given the key role of exosomes in the pathophysiology of reproduction, a better approach to their cross-talk in the female reproductive system secretome could throw some light on the development of novel diagnostic and therapeutic tools [58]. 

### 3.1. Exosomes in Polycystic Ovary Syndrome 

PCOS is a highly prevalent endocrine condition affecting 6% to 8% of women worldwide. It is characterized by ovulatory dysfunction and hyperandrogenism, which can confer a higher risk of female infertility [61,62,63,64]. In addition to reproductive abnormalities, PCOS is also linked to obesity, insulin resistance, dyslipidemia, diabetes, and cardiovascular diseases. Nevertheless, the underlying mechanism of PCOS pathogenesis is not fully elucidated [65].

To identify the role of exosomal RNAs and proteins during PCOS progression, several studies have been performed on human samples and different experimental models. For instance, a study showed a higher expression level of miR-25-3p, miR-143-3p, miR-193b-3p, miR-199a-5p, miR-199a-3p, miR-199b-3p, miR-629-5p, miR-4532, miR-4745-3p, and miR-6087 and a lower expression level of miR-10a-5p, miR-23b-3p, miR-98-5p, miR-141-3p, miR-200a-3p, miR-200c-3p, miR-382-5p, miR-483-5p, miR-483-3p, and miR-3911 in exosomes derived from human follicular fluid (HFF) in PCOS patients. These miRNAs were related to pathways of different amino acid metabolism. They also showed that a variety of tRNAs and piwi-interacting RNAs (piRNAs) were differentially expressed in HFF exosomes from PCOS patients [66]. PiRNAs are small non-coding RNA molecules that are abundant in the germline cells of animals [67]. 

Another study demonstrated that circulating exosomes in PCOS follicular fluid had differential miRNAs expression. They reported that the expression levels of miR-146a-5p and miR-126-3p were increased, and the expression levels of miR-20b-5p, miR-106a-5p, and miR-18a-3p were decreased in PCOS patients compared to healthy female controls. The differential expression of these miRNAs has been proposed to target functions, including the MAPK signaling pathway, axon guidance, circadian rhythms, endocytosis, and tumorigenesis pathways. Therefore, these exosomal miRNAs may confer a risk of PCOS [68]. Another study investigated the effect of exosomal miR-323-3p extracted from adipose mesenchymal stem cells (AMSCs) on cumulus cells (CCs) of PCOS patients. They reported that miR-323-3p inhibited apoptosis through directly targeting programmed cell death protein 4 (PDCD4) in exosome-treated CCs and thus, it alleviated PCOS [69]. More importantly, a recent study revealed that derived exosomes from PCOS patients’ serum had a significant stimulatory effect on migration and invasion of endometrial cancer cell lines. They observed differential expression of 55 miRNAs in serum exosomes from PCOS patients, among which miR-27a-5p had the most induced levels. SMAD4 is a tumor suppressor gene in the TGF-β signaling pathway and was recognized as a direct target of miR-27a-5p in this study [70].

The bioinformatics analysis exposed 245 down-regulated and 167 up-regulated circular RNAs (circRNAs) related to inflammatory pathways and oxidative stress in PCOS patients [71]. Additionally, a recent study reported that HFF exosomes from PCOS patients had a different expression level of circRNAs, among which hsa-circ-0006877 was processed from its parental low-density lipoprotein receptor (LDLR) gene, and its depletion was evaluated to be connected to a higher expression of miR-1294 and a lower expression of cytochrome P450 family 19 subfamily A member 1 (CYP19A1). In this manner, a competing endogenous RNA (ceRNA) network of hsa-circ-0006877-miR-1294-CYP19A1 may control some PCOS-related pathways, such as ovarian steroidogenesis [72]. The communication between RNAs for controlling each other’s expression through competing for shared sequences in miRNAs is recognized as ceRNA hypothesis [73]. It should be considered that specific non-coding RNAs expression correlated with PCOS development may help in an epigenetic recognition of the pathophysiology of the disease.

Previous studies highlighted the role of other exosomal markers in the development of PCOS. For example, the level of exosomal DENND1A.V2 RNA was higher in urine from PCOS patients. Furthermore, the expression level of DENND1A.V2 protein was higher in PCOS theca cells [74]. DENND1A encodes a protein linked to the clathrin-binding domain in the process of endocytosis. The DENN domain of DENND1A acts as a guanine nucleotide-exchange factor for Rab. Since Ras-related protein Rab-5B (RAB5B) interacts with the DENN domain and contributes to the insulin and MAPK signaling pathways, then it is expected that DENND1A.V2 may influence insulin or luteinizing hormone (LH)-receptor turnover and further affect ovarian function in PCOS patients [75,76]. 

Another study reported that the level of S100-A9 protein was higher in the exosomes of PCOS patients. However, they did not observe any difference in the supernatant of follicular fluid. This implied that S100-A9 acts through exosomes in follicles during PCOS development [77]. S100-A9 belongs to the calcium-binding proteins family and participates in the cell cycle, survival, proliferation, and inflammatory pathways. This protein is secreted by ovarian cells, granulosa cells, and leucocytes [78,79]. Interestingly, this study indicated that S100-A9-enriched exosomes dramatically activated the NF-κB signaling pathway and induced the expression levels of pro-inflammatory factors in a steroidogenic human granulosa-like tumor cell line (KGN) [77]. Due to the inevitable association between PCOS and inflammation [61,62], this occurrence provokes reproductive dysfunctions and PCOS progression [77]. 

Putting these findings together, exosomes play an important role as extracellular regulators in the pathophysiology of PCOS by transporting regulatory RNAs and proteins. However, further research is required for a better understanding of the exosomal cargos as potential therapeutic targets.

### 3.2. Exosomes in Premature Ovarian Failure 

POF is caused by follicular dysfunction, and the clinical manifestations are hypergonadotropism, amenorrhea, and estrogen deficiency, followed by infertility. It is reported that about 1% of all women aged 30–39 have POF [80,81]. Nevertheless, its prevalence has shown a growing tendency in recent years [82]. POF is a heterogeneous disease affected by both genetic and environmental factors; however, the exact etiology of POF is not yet fully recognized [83].

Today, stem cell therapy is regarded as a research hotspot in the field of reproductive disorder treatment, particularly POF. Hence, reviewing the related studies may offer a new approach for treating reproductive disorders and their associated infertility [84,85,86]. In this manner, most studies revealed that exosomal stem cells have an essential role in this process. For instance, a study indicated that using exosomes derived from bone mesenchymal stem cells (BMSCs) improved the follicular morphology of POF mice and suppressed apoptosis. This effect was mediated by miR-664-5p, as the main RNA in these exosomes, through targeting p53 [87]. Another study also revealed that exosomes derived from BMSCs were able to inhibit apoptosis and improve POF rats by delivering exosomal miR-144-5p and targeting PTEN [88]. 

Human amniotic epithelial cells (hAECs) are another type of stem cells applied in POF therapy. Remarkably, it was reported that hAEC-derived exosomes restored ovarian function in POF mice by transferring miR-1246 and targeting genes in the phosphatidylinositol and apoptosis pathways [89]. Furthermore, amniotic fluid stem cells (AFSCs)-derived exosomes inhibited ovarian follicular atresia in POF mice by delivering exosomal miR-10a and miR-146a, thereby regulating their target genes, including Bim, Irak1, and Traf6 in the apoptotic pathway [90]. A recent study also showed that placenta-derived mesenchymal stem cells (PD-MSCs) treatment improved ovarian function by up-regulating the expression of antioxidant enzymes, including catalase and peroxiredoxin (PRDX1) in the serum exosomes of ovariectomized rats. These enzymes contribute to mitochondrial function and decrease apoptosis by reducing reactive oxygen species (ROS) levels in the mitochondria of follicles [91]. In summation, it seems that stem cells are a novel promising therapy for POF, possibly due to the exosomal markers they represent.

### 3.3. Exosomes in Asherman Syndrome

Asherman syndrome is an acquired disorder characterized by intrauterine adhesions and clinical manifestations, such as hypomenorrhea and infertility. In this disease, adhesions form in the uterus because of trauma [84]. These scar tissues prevent the implantation of the blastocyst and cause infertility. It is reported that most Asherman syndrome patients are due to pregnancy-associated curettage. Indeed, Asherman’s syndrome has become a growing issue with the rise of cesarean and endometrial surgeries. Although this disorder can often be cured with surgery, there is still a need to develop a more practical and convenient therapy [92,93]. 

A very recent study reported that exosomal treatment might demonstrate some benefits in Asherman’s syndrome. In this study, mesenchymal stem cells (MSCs) were used to investigate the effect of exosomal MSCs on rats with Asherman syndrome. They observed that fibrosis was decreased and proliferation and vascularization were induced in uterine tissue. The expression levels of matrix metalloproteinase-2 (MMP-2), MMP-9, proliferating cell nuclear antigen (PCNA), cluster of differentiation 31 (CD31), and vascular endothelial growth factor receptor-1 (VEGFR1) were higher, and the expression level of a tissue inhibitor, metalloproteinase-2 (TIMP-2). was lower in the uterine-derived MSCs-exosomes group compared to the control group [94]. Therefore, it seems that exosomal MSCs treatment can improve the damage caused by Asherman syndrome.

### 3.4. Exosomes in Endometriosis

Endometriosis is a common multifactorial gynecological and estrogen-dependent disorder defined as the proliferation of endometrial tissue outside the uterine cavity. The distribution of endometrial cells usually involves the pelvic peritoneum, the ovaries, and the uterosacral and broad ligaments. Its severe symptoms are usually pelvic pain and infertility [95,96,97]. Considerably, endometriosis involves approximately 6-10% of all women in the world and is recurrent and refractory because of its hormone-dependence. Currently, there are no practical therapies to either cure or provide remission of endometriosis clinical manifestations. Surgery is regarded as the only treatment for advanced cases due to the lack of available tools to diagnose or treat patients in the early stages [98,99].

By RNA sequence, it was revealed that there are at least 1449 mRNAs, 938 lncRNAs, and 39 miRNAs with differential expression patterns in exosomes derived from eutopic endometrial cells, ovarian endometriomas, and normal endometrial stromal cells. Among them, 61 competing endogenous RNAs (ceRNAs) were also reported [100]. Additionally, a very recent study suggested that exosomal miR-22-3p and miR-320a with a significantly higher level in the serum of endometriosis patients might be considered available biomarkers for endometriosis diagnosis [101]. These novel molecules may open up new windows for the diagnosis of endometriosis.

Today, exosomes are significant for endometriosis, as endometrial epithelial cell-derived exosomes carry molecules with targets important in embryo–endometrial interaction during implantation [101]. Moreover, the seeding endometrial cells in endometriosis patients present epigenetic and structural alterations, and importantly, the exosomes from endometrial cells might prime the soil for attachment in ectopic areas by local regulation of cells. Consequently, retrograde menstrual cells might be implanted in this soil and create temporary lesions. Therefore, the establishment of endometriosis is facilitated [102,103,104,105]. 

Interestingly, it was reported that during the implantation period, exosomal absorption induced the trophoblast adhesion capacity. The focal adhesion kinase (FAK) pathway is the major mediatory route of this occurrence [106]. Furthermore, according to previous studies, endometrial exosomes taken by trophoblast cells have some important proteins and miRNAs that eventually augment the adhesion capacity of the trophoblast cells by modifying the expression of surface receptors contributing to adhesion. These molecules ultimately control trophoblasts’ status, such as their remodeling, migration, and adhesion capacity, all of which are essential to stabilize implantation [107,108]. Indeed, as mentioned before, miRNAs can be transferred by exosomes; among 222 miRNAs in a study, 13 miRNAs with higher levels of miR-17, miR-106a, and miR-200c were transferred by exosomes, and by bioinformatics analysis, it was demonstrated that these miRNAs played a key role in implantation [22,107]. In addition, a study implied that exosomal miR-30d is in free mode, taken up by the embryo from the endometrial fluid as a liquid full of essential nutrients to regulate the adhesion of the transcriptome and embryo [109]. Also, a recent study implied that exosomal miRNAs, including hsa-miR-494-3p, hsa-miR-10b-3p, hsa-125b-2-3p, and hsa-miR-1343-3p derived from endometrial stromal cells of endometriosis patients had higher levels and were predicted to target homeobox A10 (HOXA10) and leukemia inhibitory factor (LIF) genes related to endometrial receptivity [110].

On the other hand, there are a large amount of proteins in the exosomes regulating different signaling pathways as well as cell adhesion. These are CD47, Claudin 3, Cadherin EGF LAG Seven-Pass G-Type Receptor 2 (CELSR2), and Alpha-Parvin protein-coding gene (PARVA), which are linked to the cell polarity, and ADAM metallopeptidase domain 10 (ADAM10) and ADAMTS15, which are related to cell adhesion [108]. Another study investigated the exosomes derived from peritoneal fluid samples based on endometriosis disease stage and cycle phase. They reported the existence of some specific exosomal proteins as biomarkers, such as histone H2A type 2-C, PRDX1, inter-α-trypsin inhibitor heavy chain H4 (ITIH4), annexin A2 (ANXA2), and the tubulin α-chain, in patients that were absent in healthy individuals [111].

Several studies suggested that endometrial exosomes derived from endometriosis patients might also be important in endometriosis clinical manifestations as a disorder [102,103,104,105]. A recent study reported that exosomes collected from tissues and plasma samples of patients with endometriosis represent unique signatures of miRNAs and lncRNAs contributing to endometriosis progression. In the study, lncRNA-miRNA–seq analysis exhibited a complicated lncRNA-miR375, miR-30d-5p, and miR-27a-3p axis network, which involved a lower level of lncRNAs LINC00293, LINC00929, MEG8, SNHG25, and RP5-898J17.1 and a higher level of lncRNAs LINC00998, NEAT1, PVT1, H19, and RP4-561L24.3 in exosomes derived from ectopic endometriotic lesions. Together, these non-coding RNAs in this axis regulated many signaling pathway target genes associated with endometriosis, as well as angiogenesis and inflammation [112]. Another study documented that there is an intricate association between exosomal miRNAs, including miR-130b, miR-145, miR-342, miR-365, miR-425, miR-432, miR-451a, miR-486-5p, miR-505, miR-1908, miR-4488, and miR-6508, and inflammation in endometriosis patients [113].

Currently, the use of exosomes drew scientists’ attention to improving the endometriosis lesions through their relationship with immune responses, such as the role of macrophages in the progression of the lesions. In this case, M2 macrophages could heal these lesions by their regenerative features. In this manner, it was shown that miR-223 is the most abundant miRNA in macrophage-derived exosomes and is dysregulated in endometriosis patients. It was revealed that this miRNA contributes to the activation of M2 macrophages [114,115]. Moreover, a study by Wu et al. suggested that the transmission of exosomal miR-214 obtained from the stroma-ectopic cells resulted in fibrosis suppression and improvement of endometriosis lesions [116]. Furthermore, it was documented that exosomal miR-214-3p expression was lower in endometriosis ectopic lesions and stromal cells. They reported that exosomal miR-214-3p suppresses endometriosis fibrosis by regulating connective tissue growth factor (CCN2) as an important factor in fibrogenesis [117]. Another study indicated that ecto-nucleotidases containing exosomes in aspirates from endometriomas inhibit local immune responses required for the disease development through modulating extracellular ATP and rising extracellular adenosine levels [118]. In consequence, some exosomes might represent a practical effect on endometriosis development.

Interestingly, it was reported that exosomes from endometrial stromal cells were able to activate macrophages to be polarized into an M2-like phenotype and then enhance the progression of endometriosis lesions in mice [119]. In addition, peritoneal macrophage-derived exosomal miR-22-3p also participated in cell proliferation, migration, and invasion of ectopic endometrial stromal cells by regulating the SIRT1/NF-κB signaling pathway [120]. 

It was reported that exosomes from endometriotic stromal cells could exert enhanced angiogenic effects in vitro [121]. Indeed, it seems likely that endometrial cell-derived exosomes might be flushed retrograde into the pelvic area or be shed there by menstrual cells and affect ectopic tissues. Hence, exosomes are possible critical molecules that provoke an endometriotic lesion and produce an adequate blood supply for growing in ectopic areas as they function in intercellular crosstalk [121,122]. Remarkably, a study reported that an exosomal angiogenic-related lncRNA named antisense hypoxia-inducible factor (aHIF) was up-regulated in ectopic endometria and serum exosomes from endometriosis women. Furthermore, they observed that exosomes derived from aHIF high expression endometriotic cyst stromal cells (ECSCs) enhanced angiogenesis in human umbilical vein endothelial cells (HUVECs) through stimulating VEGF-A, VEGF-D, and basic fibroblast growth factor [123]. Another study revealed that exosomes derived from eutopic endometrium are able to promote neuroangiogenesis and enhance endometriosis [124].

All these findings suggest that exosomes could control immune evasion, cell proliferation, angiogenesis, and invasion of the lesions and subsequently regulate the development of endometriosis. In summation, intercellular crosstalk regulated by exosomes could also imply a missing connection between the different concepts on the progression of endometriosis. Exosomes derived by eutopic, ectopic, or shed endometrial tissue might lead to metaplasia of cells in ectopic areas or tissue repair after injury through their particular characteristics and also by mediating different signaling pathways [122].

### 3.5. Exosomes in Endometrial Cancer

Endometrial cancer is the fourth leading cause of malignancy of the female genital tract in women from all over the world. The incidence of endometrial cancer is growing in recent years, particularly in Europe [125]. The tumor originates from the endometrium with an abnormal proliferation of cells that have the ability to migrate and invade other parts of the body. While most patients with endometrial cancer are diagnosed early because of symptomatic postmenopausal metrorrhagia, approximately 20% of the injuries develop a high-stage tumor. Importantly, the rate of survival in these patients declines to 15%. Although surgery is suggested as the primary therapy, patients might also be treated with adjuvant radiotherapy and chemotherapy [126,127]. Therefore, there is a critical challenge to identify new targets and biomarkers as practical tools to manage endometrial cancer.

The interest in identifying circulating exosomes in a variety of biological fluids of patients with various cancers is continuously increasing. Moreover, it is believed that cancer cells secrete more exosomes than normal cells. Today, there is a great effort to explore the role of exosomes in the pathogenesis of endometrial cancer [128]. Interestingly, it is suggested that there is a cell-to-cell interaction between endometrial fibroblasts and endometrial cancer cells via exosomes carrying different regulatory RNAs [129]. In this manner, a study showed that cancer-associated fibroblasts (CAFs)-derived exosomes induced endometrial cancer progression partially due to the loss of miR-148b in the exosomes, which is an important tumor suppressor by targeting DNA (cytosine-5) methyltransferase 1 (DNMT1) to suppress endometrial cancer metastasis. DNMT1 enhances metastasis through increasing epithelial-mesenchymal transition (EMT) [130]. Moreover, another study observed that exosomal miR-320a derived from CAFs had a lower expression in endometrial cancer cells and tissues. They found out that miR-320a targets HIF1α which leads to lowered VEGFA expression and, thus, inhibits cell proliferation [131]. In contrast, exosomes, derived from plasma of patients with endometrial cancer, induced cell growth and human umbilical vein endothelial cell (HUVEC) angiogenesis through stimulation of the PI3K/AKT/VEGFA signaling pathway. In this manner, the level of plasma exosomal lectin galactoside-binding soluble 3 binding protein (LGALS3BP) was higher and was associated with VEGFA expression [132]. 

A recent study showed that endometrial cancer cells stimulated the transformation of monocyte THP-1 cells to M2-like polarization macrophages through carrying exosomal miRNA-21 in hypoxic conditions [133]. Moreover, as mentioned above, it was reported that derived exosomes from PCOS patients’ serum induced the migration and invasion of endometrial cancer cell lines. Interestingly, miR-27a-5p targeting SMAD4 had the highest induced level in these exosomes [70]. In addition, 114 dysregulated miRNAs were reported in the peritoneal lavage isolated from endometrial cancer patients using the Taqman OpenArray technology, among which miRNA-10b-5p, miRNA-34b-3p, miRNA-34c-5p, miRNA-34c-3p, miRNA-449b-5p, miRNA-200b-3p, miRNA-383-5p, and miRNA-2110 were suggested as the best biomarkers of endometrial cancer with an area under the receiver operating characteristic curve (AUC) value above 0.90 [134]. 

The exosomal hsa-miR-200c-3p was the most important biological miRNA in the urine from endometrial cancer patients, which was introduced as a non-invasive biomarker [135]. A bioinformatics study of endometrial cancer indicated that the down-regulation of Forkhead Box L2 (FOXL2) in endometrial cancer tissues or cells is associated with cell growth. When this study isolated exosomes from the supernatants of endometrial cancer cell lines, it was indicated that miR-133a targeting FOXL2 could be delivered to normal endometrial cells by exosomes [136]. Another study also observed 209 up-regulated and 66 down-regulated circRNAs in the extracellular vesicles isolated from the serum of endometrial cancer patients in stage III. The main pathway through which these circRNAs function was sequestering cancer-mediated miRNAs. More importantly, among these circRNAs, hsa circ 0109046 and hsa circ 0002577 reached a fold-change larger than two using real-time quantitative PCR [137].

Another study indicated a higher level of total (TF+), endothelial (CD144+), and monocytic (CD14+) microparticles as candidate biomarkers in peripheral and uterine blood samples of endometrial cancer patients. These results also correlated with the histologic grade and clinical staging of cancer [138]. Altogether, these studies provide novel insights into the pathogenesis of endometrial cancer considering the role of exosomes. Nevertheless, more research will be needed to explore which kind of fluid samples would give better information for diagnostic goals and target therapies in endometrial cancer management. 

### 3.6. Exosomes in Cervical Cancer

Cervical cancer, as the second leading cause of cancer death in young women, originates from the squamocolumnar junction cells of the cervix. Approximately all cases are linked to human papillomavirus (HPV) as the most common sexually transmitted infection [139,140]. Effective early screening tools might trigger early detection and prevention of the disease progression [141]. Hence, the investigation of potential biomarkers for cervical cancer is of great importance for early diagnosis and early intervention.

The first study reporting the contribution of exosomes in HPV pathogenesis was in 2009, when the existence of extracellular survivin within exosomes was confirmed in cervical carcinoma HeLa cells. These cells had anti-apoptotic and pro-proliferative features [142]. The cargo contents of HeLa-derived survivin-positive exosomes were investigated, and a total of 52 differentially expressed miRNAs were reported, among which 23 of them were affected by E6/E7 silencing. Up-regulated miRNAs had anti-apoptotic and pro-proliferative effects, while down-regulated ones had the opposite activities [143]. 

At the present time, several studies suggest the important role of different exosomal miRNAs in the progression of cervical cancer. Among them, previous studies reported a higher level of miR-21 and miR-146a in the cervicovaginal lavage specimens of cervical cancer patients [144], a higher level of let-7d-3p and miR-30d-5p, a lower level of miR-125a-5p in plasma samples of cervical cancer patients [145,146], and also a higher level of miR-221 and miR-222 in cervical cancer cell lines [147,148,149]. Importantly, miR-221-3p is capable of regulating EMT in cancer cells. It is a crucial factor in the control of local angiogenesis. Moreover, bioinformatics analysis predicted that thrombospondin-2 (THBS2) might be a direct target gene of miR-221-3p. THBS2 plays an important role in angiogenic activity [150]. Interestingly, the administration of exosomes containing high levels of miR-22 was proposed as a probable drug delivery strategy for cervical cancer radiotherapy. It is shown that miR-22 could lower the levels of c-Myc binding protein (MYCBP) and human telomerase reverse transcriptase (hTERT) [151].

Other studies have exposed the presence of lnRNAs within cervicovaginal lavage-derived exosomes, serum-derived exosomes, or HeLa-derived exosomes, including CCNDA1, HOTAIR, TUG1, MALAT1, MEG3, GAS5.132, EXOC7, lincRNA-p21, and HNF1A-AS1, as a ceRNA for miR-34b. These exosomal lncRNAs are involved in cancer progression and might have great potential to be noninvasive biomarkers for the early diagnosis of cervical cancer [152,153,154,155].

Moreover, some studies showed the presence of other molecules in exosomes derived from different cervical cancer experimental models. For example, there was a higher level of Hedgehog signaling pathway targets, including Patched1, Smoothened, Sonic hedgehog, and Indian hedgehog, in exosomes of cervical cancer cell lines. Accordingly, the Hedgehog signaling pathway plays an important role in the growth, metastasis, invasion, and drug resistance of cervical cancer [156]. Furthermore, there was a higher level of activating transcription factor 1 (ATF1) and RAS in tumors of the cervical cancer mouse model [157]. ATF1 plays a key role in cell growth, survival, and other cellular functions [158]. Also, RAS proteins are small GTPases important for mechanisms related to growth factor receptors and thus, are necessary for proliferation, and differentiation [159].

Considering the different contents of exosomes and their various activities, further investigation is needed to analyze the exosomes cargo in cervical cancer and to develop new strategies based on using exosomes for diagnostic and therapeutic purposes.

### 3.7. Exosomes in Ovarian Cancer

Ovarian cancer is among the most common types of malignant tumors in the female reproductive system and is the leading cause of gynecologic cancer deaths in the world [160]. More than 50% of patients with ovarian cancer are in an advanced stage when they are referred to clinics. Each year, more than 230,000 new patients and 150,000 deaths due to ovarian cancer are reported all over the world. Remarkably, the 5-year survival rate for patients is less than 50% [125,161]. The poor survival rates and low quality of life for patients are partly due to the lack of early diagnostic tools. Hence, developing more practical applications in the diagnosis and treatment of the disease is essential to prevent the rise of disease incidence [162]. 

Exosomes excreted from ovarian cancer cells could be up-taken by other tumor or normal cells to increase intercellular interaction linked to tumor development, metastasis, and invasion. Moreover, exosomes derived from ovarian cancer could serve as novel biomarkers and therapeutic targets [161]. Here, we aim to summarize the results of some of the studies regarding the role of exosomes in the pathology of ovarian cancer.

A large number of proteins are recognized in or on ovarian cancer-derived exosomes. Some of these proteins are involved in the malignant behavior of the tumor. Indeed, exosomes communicate with other cells and act as vehicles for transferring different proteins among cells. In this context, proteins might affect cell signaling or change the tumor microenvironment in a way that induces tumor growth and metastasis [163,164]. 

For instance, it is indicated that membrane proteins, such as TSG 101 and Alix; as well as tetraspanins; including CD9, CD24, CD44, and CD63, transferred by exosomes contribute to the development of ovarian cancer. Furthermore, it is reported that exosomal Hsp70 and Hsp90 are involved in the pathogenesis of the disease [165,166,167,168]. Interestingly, a study revealed a higher expression of Hsp27 in the exosomes of patients with ovarian cancer [169]. 

Other exosomal proteins introduced as critical factors in ovarian cancer are enzymes (aldehyde reductase, phosphate isomerase, fatty acid synthase, and peroxiredoxin) and antigens (MHC I and II) [162,170]. These factors are either related to tumor development or metastasis. Noticeably, a very recent study showed the higher concentration of lipoprotein lipase (LPL) and collagen type V alpha 2 chain (COL5A2) in exosomes derived from ovarian cancer cells (SKOV-3) compared to ovarian surface epithelial cells (HOSEPiC) by proteomic and lipidomic analysis [171]. Furthermore, exosomal proteins might be involved in drug resistance. For instance, annexin A3 is an exosomal protein secreted from cisplatin-resistant cells, and its higher expression is linked to platinum resistance in cancer cells [172]. 

On the other hand, researchers have started to investigate the relationship between exosomal miRNAs and their influence on the pathogenesis of ovarian cancer. Previous studies have revealed that exosomes could change the chemo-susceptibility in recipient cells by regulating different biological pathways, including cell cycle and apoptosis. For instance, miR-106a, miR-130a, miR-221, miR-222, miR-433, and miR-591 are introduced as modulators of drug resistance in ovarian cancer [173,174,175,176,177]. Additionally, a recent study indicated that macrophage-derived exosomes transfer miR-223 to epithelial ovarian cancer cells to promote drug resistance through the PI3K/AKT signaling pathway [178].

Previous studies suggested miR-200f as a diagnostic marker since the level of miR-200f is increased in the circulation of epithelial ovarian carcinoma patients [179,180,181]. In addition, a recent study reported a higher expression of exosomal miR-21, miR-100, and miR-320 and a lower expression of miR-16, miR-93, and miR-126 in the plasma of patients with epithelial ovarian carcinoma [182]. Additionally, other recent investigations revealed the role of epithelial ovarian carcinoma-derived exosomal miRNAs, including miR-141-3p and miR-205, in stimulating the vascularization of endothelial cells [183,184].

Accordingly, exosomal miRNAs, such as miR-21, miR-184, miR-193b, miR-200a, miR-200b, miR-200c, miR-203, miR-214, and miR-215, could be regarded as diagnostic biomarkers [168,170,185,186,187]. Other exosomal miRNAs also contribute to tumorigenesis and invasion. For instance, let-7 miR, miR-21, miR-25, miR-29b, miR-100, miR-105, miR-150, miR-187, miR-221, and miR-335 are reported to be involved in the development of malignant ovarian tumors [168,170,188]. Among them, miR-21 is shown to play an important role in oncogenesis and metastasis through targeting PDCD4 as a tumor suppressor in serous ovarian carcinoma [189]. Other miRNAs, including miR-29c, miR-101, miR-128, miR-182, miR-506, and miR-520d-3p, are also suggested as therapeutic targets for ovarian cancer treatment [190].

Altogether, these studies suggest that different non-coding RNAs and proteins with distinct roles are important exosomal cargos in ovarian cancer that alter the biology of the disease and could be regarded for diagnosis and treatment. Nevertheless, more investigation is required to fully describe the effect of exosomes on the malignant activity of ovarian cancer. 

### 3.8. Exosomes in Preeclampsia

Preeclampsia is a hypertensive pregnancy abnormality associated with maternal and fetal mortality causing 10-15% of all fetal deaths if not diagnosed and treated promptly. It usually happens after 20 weeks of pregnancy due to placental hypoxia resulting in deficient spiral artery remodeling [191,192,193,194]. While preeclampsia is characterized by multifaceted communications between maternal and placental factors and insufficient spiral-artery remodeling mediated by trophoblast invasion, a comprehensive prospect for the pathogenicity of the syndrome remains unclear [195,196]. On the other hand, although several biomarkers have been introduced for preeclampsia, they have been confirmed to be unsuccessful in providing a decisive diagnosis during the different stages of the syndrome [197].

As mentioned before, cell–cell communication plays an important role in feto-placental development in healthy pregnancies [198]. Interestingly, since the placenta plays an important role in the pathology of preeclampsia, it is tempting to speculate that a higher release of exosomes into the maternal circulation by the placental trophoblasts is a feature of the disorder. Indeed, the production and release of placental-derived exosomes are induced during pregnancy as well as in other complications of pregnancy, such as preeclampsia [199]. Furthermore, it is revealed that augmented placental oxygen tension caused by a predisposing situation increases the release of exosomes from the syncytial layer of the placenta [200,201]. Therefore, the placental-derived exosomal profile might play a critical role in identifying women with preeclampsia.

Several studies indicated different immunological and metabolic functions of exosomes related to preeclampsia [202,203]. Among the protein content of placental trophoblast-derived exosomes is syncytin, which might be involved in the syncytiotrophoblast formation from villous trophoblasts. These trophoblasts invade spiral arteries and transform the maternal vascular endothelial and smooth muscle cells [204,205]. Accordingly, higher circulating levels of syncytiotrophoblast-derived exosomes have been observed in patients with preeclampsia [206]. Additionally, a study showed that exosomal syncytin-2 levels are considerably lower in the circulation of patients with preeclampsia [205]. 

Previous studies also reported that tissue factor is expressed on the surface of syncytiotrophoblast-derived exosomes [207,208]. Tissue factor is a transmembrane protein that functions in the clotting process. The overexpression and higher activity of tissue factor on syncytiotrophoblasts are related to preeclampsia [209,210]. Importantly, a study indicated that using anticoagulants in an animal model of preeclampsia alleviated clinical manifestations [211]. Moreover, it is demonstrated that placental trophoblast-derived exosomes have several serine proteases and metalloproteases (MMP), such as MMP-12. Therefore, exosomal MMP-12 might help the process of trophoblasts invasion by remodeling the extracellular matrix [212,213].

The influence of other cargos of exosomal content, including miRNA, has been the focus of several studies [214,215,216]. For example, a study showed a lower expression of miR-23a-3p, miR-125b-2-3p, miR-144-3p, miR-192-5p, miR-205-5p, miR-208a-3p, miR-335-5p, miR-451a, miR-518a-3p, and miR-542-3p and a higher expression of let-7a-5p, miR-17-5p, miR-26a-5p, miR-30c-5p, miR-141-3p, miR-199a-3p, miR-221-3p, miR-584-5p, miR-744-5p, and miR-6724-5p in exosomes isolated from patients with preeclampsia compared to normal women. Noticeably, three miRNAs, including hsa-miR-525-5p, hsa-miR-526b-5p, and hsa-miR-1269b, were recognized only in the disease conditions [217]. In this manner, it should be noted that these miRNAs are important in signaling pathways related to the pathogenesis of preeclampsia. First, miR-525e5p is capable of suppressing the vasoactive intestinal peptide (VIP) as a strong anti-inflammatory factor [218]. In addition, miR-526b regulates the expression of MMP-1 and HIF-1a [219]. Finally, miR-1269 controls the expression of forkhead box O1 gene (FOXO1) as a critical factor in the endometrial stromal decidualization and the implantation process [217,220].

In summation, the ability to identify exosomes released during pregnancy and introducing non-invasive interventions to alleviate their effects seems to be of great importance in clinical use for the diagnosis and treatment of preeclampsia. However, further studies are required to recognize the underlying mechanisms that trigger the release of exosomes involved in the progression of preeclampsia.

## 4. Clinical Diagnosis and Therapeutic Approaches

A notable advantage of circulating exosomes versus other biomarkers is that these vesicles have higher amounts of materials for further analyses, such as genetic examinations. Today, exosomes might be regarded as the future of diagnostic procedures for many diseases, including reproductive disorders. These vesicles carry various molecular cargos that could be applied to diagnose such diseases as cancer without the necessity of biopsy [196].

Nevertheless, these findings of exosomes and their particular cargos in various biological fluids are still quite exploratory, and more validation in clinical investigations with standardized protocols is required before their routine use in the clinic as biomarkers with diagnostic and prognostic relevance. This is because there is much controversy concerning the isolation and detection of exosomes [221,222]. Consequently, these vesicles must be accurately collected and characterized from biological fluids to be utilized as diagnostic agents. Today, several kits have been commercialized for better and simplified isolation, including ExoQuick (System Bioscience) [128,222]. Moreover, we hope that further standardization methods would resolve these issues. Interestingly, next-generation sequencing or nanoparticle-tracking analysis are frontiers in medical technology to improve the effectiveness of exosomal collection and detection and identify novel exosomes related to reproduction-related complications [196].

Targeted therapy is another new approach that can be utilized for women with reproductive disorders by changing different factors in the major signaling pathways related to disease progression [223]. It is tempting to speculate that the future control of female-reproductive-system-related diseases would be dependent on the identification of novel targets, which in turn might be used to personalized medicine. As discussed above, exosomes are vesicles for carrying different RNAs and proteins and, during pathological conditions, the entity of circulating exosomes, particularly their production and trafficking rate, are changed [59,60]. Therefore, given the significant role of exosomes in the pathophysiology of reproduction, understanding their interaction in the reproductive system secretome could shed light on the development of novel therapeutic tools [58].

In the present, stem cell therapy has received great attention in the field of treatment of reproductive disorders due to many benefits, such as abundant sources, self-renewal, differentiation, and unnecessary ethical considerations. Many scientists suggested the use of different stem cells in the therapeutic strategies associated with female infertility or other complications. Remarkably, several studies exposed that exosomal stem cells might play a critical role in this process due to having no risk of aneuploidy and a lower possibility of immune rejection after in vivo allogeneic administration [84,224]. Indeed, previous studies indicated the potential therapeutic role of exosomal stem cells in different diseases, including immune diseases [225], cancer [226], cardiovascular diseases [224], and neurodegenerative diseases [227]. Here, the present study also hypothesized that exosomes are able to reprogram diseased cells in different diseases due to their ability to regulate target cells by carrying RNAs and proteins [228,229]. Therefore, it is likely that these molecules derived from stem cells could be considered as a novel therapy in fertility clinics.

Altogether, today, exosomes might serve as diagnostic biomarkers and therapeutic targets in pregnancy-associated disorders or placental functions, including PCOS, POF, Asherman syndrome, endometriosis, endometrial cancer, cervical cancer, ovarian cancer, and preeclampsia. Although studies on the role of exosomes in the pathophysiology of other reproductive disorders, such as uterine fibroid and leiomyosarcoma, are limited, and thus, further studies are required to investigate them. Nevertheless, the validation for introducing exosomes as potential molecules for controlling reproductive disorders is yet to be studied in terms of the FDA-approved biomarker criterion [230].

From this study we can summarize the etiology of reproductive dysfunction and improve the early diagnosis and treatment of related complications and the use of exosomes by summarizing all the data in Table 1.

## 5. Conclusions

In the present review, we summarized findings from exosome studies focusing on the main components, including different RNAs and proteins, which mediate the functions of target cells by either induction of surface ligands or transferring factors associated with various biological pathways. Remarkably, the transfer of mRNAs could result in transmitting genetic information and the synthesis of various proteins. Exosomes carry numerous functional proteins that are responsible for various physiological and pathological conditions in the target cells. Moreover, the transfer of non-coding RNAs also has a notable effect on the regulation of gene expression. Therefore, these studies hypothesize that exosomes represent ideal vesicles for carrying transcripts, proteins, and non-coding RNAs, and thus, could be regarded as diagnostic biomarkers and therapeutic targets in the field of reproductive disorders.

## Figures and Tables

**Table 1:** A summary of exosomes related to different female reproductive system diseases.

Disease	Source	Studied cargo	Type	Clinil Level	Clinical Value	References
**PCOS**	Follicular fluid	miR-25-3p, miR-143-3p, miR-193b-3p, miR-199a-5p, miR-199a-3p, miR-199b-3p, miR-629-5p, miR-4532, miR-4745-3p, miR-6087	miRNA	Up	Diagnosis and Target therapy	[66]
	Follicular fluid	miR-10a-5p, miR-23b-3p, miR-98-5p, miR-141-3p, miR-200a-3p, miR-200c-3p, miR-382-5p, miR-483-5p, miR-483-3p, miR-3911	miRNA	Down	Diagnosis and Target therapy	[66]
	Follicular fluid	miR-146a-5p, miR-126–3p	miRNA	Up	Diagnosis and Target therapy	[68]
	Follicular fluid	miR-20b-5p, miR-106a-5p, miR-18a-3p	miRNA	Down	Diagnosis and Target therapy	[68]
	AMSCs	miR-323-3p	miRNA	-	Stem cell therapy	[69]
	Serum	miR-27a-5p	miRNA	Up	Target therapy	[70]
	Follicular fluid	hsa_circ_0006877	circRNA	Down	Target therapy	[72]
	Urine	DENND1A.V2	Protein	Up	Target therapy	[74]
	Follicular fluid	S100-A9	Protein	Up	Target therapy	[77]
**POF**	BMSCs	miR-664-5p	miRNA	-	Stem cell therapy	[87]
	BMSCs	miR-144-5p	miRNA	-	Stem cell therapy	[88]
	hAECs	miR-1246	miRNA	-	Stem cell therapy	[89]
	AFSCs	miR-10a, miR-146a	miRNA	-	Stem cell therapy	[90]
	PD-MSCs	antioxidant enzymes [e.g., catalase, and PRDX1]	Protein	-	Stem cell therapy	[91]
**Asherman syndrome**	MSCs	MMP-2, MMP-9, PCNA, CD31, VEGFR1	Protein	Up	Stem cell therapy	[94]
	MSCs	TIMP-2	Protein	Down	Stem cell therapy	[94]
**Endometriosis**	Serum	miR-320a	miRNA	Up	Diagnosis	[101]
	Peritoneal macrophages	miR-22-3p	miRNA	Up	Diagnosis and Target therapy	[120]
	ECC1 cells	miR-17, miR-106a, miR-200c	miRNA	Up	Diagnosis and Target therapy	[22]
	Endometrial fluid	miR-30d	miRNA		Target therapy	[109]
	Endometrial stromal cells	hsa-miR-494- 3p, hsa-miR-10b-3p, hsa-125b-2-3p, hsa-miR-1343-3p	miRNA	Up	Target therapy	[110]
	Macrophages	miR-223	miRNA		Target therapy	[114]
	Stroma-ectopic cells	miR-214	miRNA	Down	Target therapy	[116,117]
	Ectopic endometriotic lesions	LINC00998, NEAT1, PVT1, H19, RP4-561L24.3	LncRNA	Up	Target therapy	[112]
	Ectopic endometriotic lesions	LINC00293, LINC00929, MEG8, SNHG25, RP5-898J17.1	LncRNA	Down	Target therapy	[112]
	Ectopic endometria, serum, ECSCs	aHIF	LncRNA	Up	Diagnosis and Target therapy	[123]
	Peritoneal fluid	PRDX1, ITIH4, ANXA2, tubulin α-chain	Protein	Up	Diagnosis	[111]
**Endometrial Cancer**	CAFs	miR-148b	miRNA	Down	Target therapy	[130]
	CAFs	miR-320a	miRNA	Down	Target therapy	[131]
	Endometrial cancer cells	miRNA-21	miRNA	Up	Target therapy	[133]
	Peritoneal lavage	miR-10b-5p, miR-34b-3p, miR-34c-5p, miR-34c-3p, miR-449b-5p, miR-200b-3p, miR-383-5p, miR-2110	miRNA	Down	Diagnosis	[134]
	Urine	hsa-miR-200c-3p	miRNA	Up	Diagnosis	[135]
	Supernatants of endometrial cancer cell lines	miR-133a	miRNA	Up	Target therapy	[136]
	Serum	hsa circ 0109046, hsa circ 0002577	circRNA	Up	Diagnosis	[137]
	Plasma	LGALS3BP	Protein	Up	Target therapy	[132]
	Peripheral and uterine blood	TF+, CD144+, CD14+	Protein	Up	Diagnosis	[138]
**Cervical Cancer**	Cervicovaginal lavage	miR-21, miR-146a	miRNA	Up	Diagnosis	[144]
	Plasma	let-7d-3p miR-30d-5p	miRNA	Up	Diagnosis	[145]
	Plasma	miR-125a-5p	miRNA	Down	Diagnosis	[146]
	Cervical cancer cell lines	miR-221, miR-222 in	miRNA	Up	Diagnosis and Target therapy	[147,148,149,150]
	HEK293	miR-22	miRNA	Down	Target therapy	[151]
	HeLa	HNF1A-AS1	LncRNA	Up	Diagnosis	[152]
	Cervicovaginal lavage	HOTAIR, MALAT1, MEG3	LncRNA	Up	Diagnosis	[153]
	Serum	EXOC7	LncRNA	Up	Diagnosis	[154]
	CerEpiC, HeLa, CaSki	TUG1	LncRNA	Up	Diagnosis	[155]
	Cervical cancer cell lines	Patched1, Smoothened, Sonic hedgehog, Indian hedgehog	Protein	Up	Target therapy	[156]
	Mouse cervical cancer cells	ATF1, RAS	Protein	Up	Target therapy	[157]
**Ovarian Cancer**	Serum	miR-21	miRNA	Up	Diagnosis and Target therapy	[189]
	Plasma	miR-100, miR-320	miRNA	Up	Diagnosis	[182]
	Plasma	miR-16, miR-93, miR-126	miRNA	Down	Diagnosis	[182]
	Paclitaxel-resistant ovarian cancer cell	miR-106a	miRNA	Up	Drug resistance	[175]
	Paclitaxel- and cisplatin-resistant ovarian cancer cell	miR-130a	miRNA	Down	Drug resistance	[176]
	Serum	miR-200	miRNA	Up	Diagnosis	[179,180,181]
	Ovarian cancer tissues	miR-221, miR-222	miRNA	Up	Drug resistance and Target therapy	[174]
	Macrophages	miR-223	miRNA	Up	Drug resistance	[178]
	Paclitaxel-resistant ovarian cancer cell	miR-591	miRNA	Down	Drug resistance	[175]
	Ovarian cancer cell lines	miR-433	miRNA	Down	Drug resistance	[173]
	Ovarian cancer cell lines	CD9, CD63	Protein	Up	Diagnosis and Target therapy	[168]
	Ovarian cancer cell lines	CD24	Protein	Up	Diagnosis and Target therapy	[165]
	Epithelial ovarian cancer	CD44	Protein	Up	Diagnosis and Target therapy	[166]
	Serum	Hsp27	Protein	Up	Diagnosis and Target therapy	[169]
	Dendritic cells	MHC I, Hsp70, Hsp90	Protein	Up	Diagnosis and Target therapy	[167]
	Ovarian cancer cell lines	Aldehyde reductase, phosphate isomerase, fatty acid synthase, and peroxiredoxin	Protein	Up	Diagnosis and Target therapy	[162,170]
	Ovarian cancer cell lines	LPL, COL5A2	Protein	Up	Diagnosis and Target therapy	[171]
	Cisplatin-resistant ovarian cancer cells	Annexin A3	Protein	Up	Drug resistance	[172]
**Preeclampsia**	Extravillous trophoblast cells	miR-23a-3p, miR-125b-2-3p, miR-144-3p, miR-192-5p, miR-205-5p, miR-208a-3p, miR-335-5p, miR-451a, miR-518a-3p, miR-542-3p	miRNA	Down	Diagnosis	[217]
	Extravillous trophoblast cells	let-7a-5p, miR-17-5p, miR-26a-5p, miR-30c-5p, miR-141-3p, miR-199a-3p, miR-221-3p, miR-584-5p, miR-744-5p, miR-6724-5p, * hsa-miR-525-5p, * hsa-miR-526b-5p, * hsa-miR-1269b	miRNA	Up	Diagnosis and Target therapy	[217]
	Placental trophoblast	Syncytin-2	Protein	Down	Target therapy	[205]
	Syncytiotrophoblast	Tissue factor	Protein	Up	Target therapy	[209,210]

* These three miRNAs were recognized only in the disease conditions. miR, miRNA, microRNA; PCOS, polycystic ovary syndrome; POF, premature ovarian failure; AMSCs, adipose mesenchymal stem cells; BMSCs, bone mesenchymal stem cells; hAECs, human amniotic epithelial cells; AFSCs, PD-MSCs, amniotic fluid stem cells; placenta-derived mesenchymal stem cells; peroxiredoxin; MMP-2, matrix metalloproteinase-2; PCNA, proliferating cell nuclear antigen; CD31, cluster of differentiation 31; VEGFR1, vascular endothelial growth factor receptor-1; TIMP-2, tissue inhibitor of metalloproteinase-2; aHIF, antisense hypoxia-inducible factor; ITIH4, inter-α-trypsin inhibitor heavy chain H4; ANXA2, annexin A2; CAFs, cancer-associated fibroblasts; LGALS3BP, lectin galactoside-binding soluble 3 binding protein; ATF1, activating transcription factor 1; Hsp27, heat shock protein 27; LPL, lipoprotein lipase; COL5A2, collagen type V alpha 2 chain.

## Data Availability

Not applicable.
